# HEALTHY CAREGIVER SELECTION AMONG DEMENTIA CAREGIVERS: THE ROLE OF SOCIAL SUPPORT

**DOI:** 10.1093/geroni/igac059.3117

**Published:** 2022-12-20

**Authors:** Hantao Jiang, Xueqing Wang

**Affiliations:** Columbia University, Princeton, New Jersey, United States; Princeton University, Princeton, New Jersey, United States

## Abstract

As of 2019, more than 4 million older adults aged 65+ in the United States are cognitively impaired, including the diagnoses of mild cognitive impairment (MCI) and dementia. Caregivers to these older adults bear significant burden, reflected as high prevalence of chronic stress and mental health problems among the caregiver population. It is thus crucial to understand the wellbeing of the caregiver population to design effective policies. Previous studies have documented survival advantage of dementia caregivers compared to non-caregiving individuals as well as other types of caregivers, namely caregivers to persons with MCI or other types of chronic conditions. However, it remains less clear how the role of social support explains dementia caregiver’s survival advantage. In this paper, we directly compare the level and type of social support between different types of caregivers, and examine to what extent the difference in social support explains the survival and health advantage of dementia caregivers compared to caregivers to persons with MCI, non-cognitive impairment chronic conditions and non-caregiving individuals. We use the 12 waves of the Health and Retirement Study and apply multivariate and survival analysis to calculate difference in age-specific hazard ratios. Our preliminary results show that dementia caregivers tend to secure stronger support from family members than caregivers to persons with MCI. Our results have potential to shed light on the empirical puzzle of healthy caregiver selection effect and have direct implications for designing effective intervention to improve health of the caregiver population.

